# Effects of cannabidiol on weight and fasting blood sugar with chronic and subchronic haloperidol administration

**DOI:** 10.1007/s44192-022-00021-2

**Published:** 2022-07-14

**Authors:** Jaiyeola Abiola Kajero, Soraya Seedat, Jude U. Ohaeri, Abidemi Akindele, Oluwagbemiga Aina

**Affiliations:** 1 Federal Neuropsychiatric Hospital Yaba, 8, Harvey Road Yaba, P.M.B 2008, Lagos, Nigeria; 2 Department of Psychiatry, Faculty of Medicine and Health Sciences, Stellenbosch University, Francie van Zijl Drive Tygerberg, Cape Town, 7505 South Africa; 3 Department of Psychological Medicine, University of Nigeria Teaching Hospital, Enugu, Enugu State Nigeria; 4 Department of Pharmacology, Therapeutics & Toxicology, Faculty of Basic Medical Sciences, College of Medicine, University of Lagos, Private Mail Bag 12003, Lagos, Nigeria; 5 Department of Biochemistry and Nutrition, Nigerian Institute of Medical Research (NIMR), 6, Edmund crescent off Murtala Mohammed way, Yaba, P.M.B. 2013, Lagos, 100001 Nigeria

**Keywords:** Fasting blood sugar, Body weight, Haloperidol, Cannabidiol, Metabolic syndrome

## Abstract

**Objectives:**

The duration of administration (e.g., subchronic or chronic) of haloperidol may influence its adverse effects. We studied the effects of duration of administration of haloperidol on body weight and fasting blood sugar (FBS). In addition, we examined whether orally administered cannabidiol (CBD) had any putative mitigating influence on haloperidol-induced body weight changes and FBS elevation.

**Methods:**

Haloperidol (5 mg/kg/day) was administered for 21 days (subchronic administration), via the intraperitoneal (IP) route, or monthly (50 mg/kg monthly) for 3 months (chronic administration), via the intramuscular (IM) route, either alone or before CBD (5 mg/kg/day). Oral CBD (5 mg/kg/day) alone and distilled water alone were administered for 21 days. Weight and FBS were measured before administration of pharmacological agents (distilled water in the control group) and post-administration.

**Results:**

Group differences in average weight across time were significant. Pairwise comparisons showed that mean weight of the subchronic (IP) haloperidol alone group (Group A) and the chronic (IM) haloperidol before CBD group (Group F) increased significantly over time. Post medications, there was a significant increase in mean FBS in the subchronic (IP) haloperidol group compared to the subchronic (IP) haloperidol before CBD group. There was also a significant reduction in mean FBS from the baseline for the control group only.

**Conclusion:**

We demonstrated that the duration of administration of haloperidol influenced weight and FBS in rats, suggesting that metabolic side effects, may be influenced by duration of administration. CBD ameliorated the increase in weight and FBS observed in the subchronic (IP) haloperidol groups.

**Supplementary Information:**

The online version contains supplementary material available at 10.1007/s44192-022-00021-2.

## Introduction

In both animal and human studies, chronic administration of antipsychotics has been shown to induce body weight gain, dyslipidaemia, hyperinsulinemia, and hyperglycaemia – all risk factors for metabolic syndrome [1, 2]. There is also accumulating evidence for the role of dopamine in the development of obesity. Antagonism at dopamine D_2_ receptors in the ventral tegmental area (VTA), the mechanism of action of many antipsychotics, leads to dopaminergic hypofunction, appetite stimulation, increased food intake and weight gain [3, 4]. Higher occupancy of D_2_ receptors by chronic administration of haloperidol may, therefore, lead to an increase in weight and metabolic dysfunction.

While the influence of prolonged administration of haloperidol on body weight and fasting blood sugar (FBS) has not been studied, it has been suggested that duration of administration may influence its adverse effects [5, 6]. For example, intramuscular haloperidol may elevate blood glucose in previously controlled diabetes mellitus [7]. Acute administration of a high dose of haloperidol has also been associated with elevated FBS [8].

Pharmacological agents that interact with D_2_ receptors in the brain and ß cells in the islets of Langerhans in the pancreas may be beneficial in ameliorating the adverse effects of haloperidol on body weight and FBS. CBD is one agent that influences weight and fasting blood sugar in both animals and humans, most likely through its agonist actions at 5-HT_1A_ receptors, and cannabinoid GPR55 receptors in the islets of Langerhans [9–12]. It may also act as an antagonist/ inverse agonist at CB1 receptors [13, 14]. Further, CBD is a partial agonist at dopamine receptors [15]. Several researchers have observed body weight loss with CBD [16, 17] and CBD has, in addition, been shown to induce hyperinsulinemia and reduce FBS [18]. CBD may, therefore, be a putative therapeutic agent for antipsychotic-induced metabolic syndrome. There are, to our knowledge, no published studies on the dynamics of the duration of haloperidol administration on body weight and FBS, nor on the putative modifying effects of CBD on FBS. This sub-study sought to investigate the effects of the duration of administration of haloperidol and adjunctive CBD on weight gain and FBS. The overarching parent study was designed to explore the putative beneficial effects of CBD in the management of vacuous chewing movements (VCM) in an animal model of tardive dyskinesia (TD).

## Materials and methods

### Animals

Male adult Wistar rats used in this study were obtained from the colony of the Nigerian Institute of Medical Research (NIMR), Yaba, Lagos, Nigeria. The animals were kept in clean polypropylene cages in well-ventilated and hygienic compartments, maintained under standard environmental conditions and fed with standard rodent pellets (Ladokun Feeds Plc., Ibadan, Nigeria) and water ad libitum*.* The animals were acclimatised for a period of 2 weeks before experimental procedures were undertaken, in accordance with the United States National Institutes of Health Guidelines for Care and Use of Laboratory Animals in Biomedical Research [19]. The study was approved by the Institutional Review Board (IRB) of NIMR, Yaba, Lagos, Nigeria (IRB/16/329) and Stellenbosch University’s Health Research Ethics Committee: Animal Care and Use (SU-ACUD16-00137).

### Drugs

Cannabidiol [(-)-Cannabidiol, GMP (Cannabidiolum); CBD] (VAKOS X, a.s., Permova 28a, Praha, Czech Republic) was supplied in fine granule form, with the amount administered weekly calculated and dissolved in 70% ethanol, as recommended by the manufacturer, and diluted with distilled water. CBD was administered orally. Rapid-acting parenteral haloperidol 5 mg/ml and slow-releasing parenteral haloperidol 50 mg/ml (Janssen Pharmaceuticals, Beerse, Belgium) were also administered.

### Experimental design

There were 6 experimental groups (n = 53): (1) Group A (Haloperidol 5 mg/kg i.p. only); (2) Group B (Haloperidol 5 mg/kg i.p. before administration of CBD 5 mg/kg p.o.); (3) Group C (CBD 5 mg/kg p.o. only); (4) Group D (Control (Distilled water) 2 ml p.o.); (5) Group E (Haloperidol decanoate 50 mg/kg i.m. only); and (6) Group F (Haloperidol decanoate 50 mg/kg i.m. before administration of CBD 5 mg/kg p.o.).

The administration was as follows: For Groups A, C and D, agents were administered once daily for 21 days [20–23] (Table 1). A dose of 5 mg/kg/day of haloperidol was administered in the subacute (IP) groups A and B [23]. Effective doses of CBD in rats range between 2.5 and 10 mg/kg/day [24]. We opted for 5 mg/kg of CBD, using our previous experiments as a guide [25].

**Table 1:** Pharmacological administration schedule

Group	Pharmacological agent	Administration schedule
Group A	Haloperidol 5 mg/kg i.p. only	HAL 21 days
Group B	Haloperidol 5 mg/kg i.p. before administration of CBD 5 mg/kg p.o.)	HAL for 21 days then CBD for 21 days
Group C	CBD 5 mg/kg p.o. only	CBD 21 days
Group D	Control (Distilled water) 2 ml p.o	21 days
Group E	Haloperidol decanoate 50 mg/kg i.m. only)	HAL DECAONATE monthly for three months
Group F	Haloperidol decanoate 50 mg/kg i.m. before administration of CBD 5 mg/kg p.o	HAL DECAONATE monthly for 3 months then CBD for 21 days

For Group B, the first pharmacological agent (Haloperidol 5 mg/kg i.p.) was administered for 21 days and the second agent (CBD 5 mg/kg p.o.) was commenced 24 h after completion of administration of the first agent and administered for another 21 days. Groups E and F constituted the chronic haloperidol administration groups. For Group E, IM haloperidol decanoate 50 mg/kg only was administered monthly on three consecutive occasions [26]. FBS was measured 21 days after the completion of IM haloperidol decanoate 50 mg/kg administration. For Group F, IM haloperidol decanoate 50 mg/kg was administered monthly on three consecutive occasions, followed by administration of cannabidiol 5 mg/kg for 21 days. CBD administration was commenced 24 h after the last dose of IM haloperidol (i.e., after the 3rd dose) (Table 1).

#### Fasting blood sugar (FBS) measurement

The rats were fasted overnight after the last meal at 7 pm until 7am the following day when blood was taken for FBS measurement. A fine sterile disposable hypodermic needle was used to puncture the rats at the end of the tail and enough blood was dropped on a portable glucometer device, Accu-Chek Performa® (Roche Diagnostics, Mannheim, Germany) to read the FBS.

#### Weight measurement

An electronic weighing scale designed for rats/mice was used to measure weight. An appropriate beaker was placed on the electronic weighing scale. The scale was tared back to 0, and the bowl taken off, and each animal was placed gently in the bowl and placed on the scale to record the weight.

Weight and FBS were measured before administration of pharmacological agents and at the end of the administration of all agents. For both Groups E and F, weight, FBS and VCM were measured at the same time to eliminate the effect of normal growth. An automated weighing scale was used to dispense and monitor the average amount of food to the animals per housing box.

### Statistical analysis

Data were analysed using IBM SPSS Statistics for Windows, Version 28.0 (Armonk, NY: IBM Corp). A mixed-effect model, which assumes that the explanatory variables have different relationships with the response variable within groups (e.g., subjects) but share the same fixed relationship across groups, was used to analyse data on weight in grams and FBS, with treatment and time as two fixed effects and rats’ weight and FBS as random effects. Weight and FBS differences within and across groups were observed.

## Results

### Effect on weight gain

Using a mixed-method ANOVA, we found significant changes in mean weight from baseline when we compared the pre- (mean baseline weight) and post-medication mean weight difference in each group with other groups (F = 4.505, p = 0.002). Post-hoc analysis revealed a significant difference between Groups B and F (p = 0.035), D and F (0.046) (Table 2).

**Table 2:** Group comparisons of mean weight change from baseline

Group	N	Mean weight change	Std. Error	Range of changes		
				Minimum	Maximum	
A	9	23.22	13.844	− 43	84	
B	10	4.30	6.539	− 17	36	F = 4.505 p = **0.002**
C	9	10.78	5.946	− 8	41	
D	10	3.20	12.147	− 38	97	
E	8	20.50	9.703	− 12	64	
F	7	69.57	15.388	31	138	

**Fig. 1:**
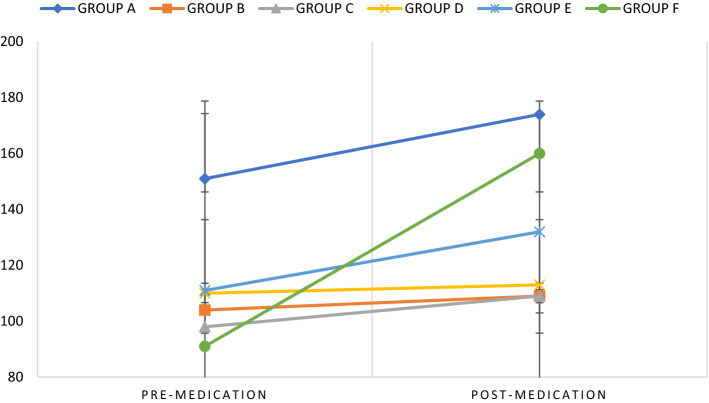
Within Subject Effects: Estimated Marginal Means of weight over time. *Standard deviations are represented as black dashes (–) on the black vertical lines connecting the coloured lines

At baseline, the mean weight in Group A differed significantly from all groups (B, C, D, E, F) and at post-administration, the mean weight in Group A differed significantly from four other groups (B, C, D, E) but not from Group F. Also, the mean weight of Groups B, C, D differed significantly from Group F. When the mean baseline weight (pre-medication) of each group was compared with the post-medication weight of each group, the mean weight changed significantly only in Group A (p = 0.036) and F (p < 0.001). The mean weight of groups A and F increased significantly over time; however, the mean weight of Groups B, C, D and E did not change significantly over time (Fig. 1).

Group A: Haloperidol 5 mg/kg i.p., Group B: Haloperidol 5 mg/kg i.p. before Cannabidiol 5 mg/kg p.o., Group C: Cannabidiol 5 mg/kg p.o., Group D: Control (Distilled water) 2 ml p.o. Group E: Haloperidol decanoate 50 mg/kg i.m., Group F: Haloperidol decanoate 50 mg/kg i.m. before administration of cannabidiol 5 mg/kg p.o.

### Pairwise comparisons

Pre-medication mean weight in each group revealed a significant between-group difference for Groups A > B (p < 0.001), A > C (p < 0.001), A > D (p < 0.001), A > E (p = 0.004), A and F (p < 0.001) (Fig. 1).

Post-medication mean weight across groups revealed a significant between-group difference for Groups A > B (p < 0.001), A > C (p < 0.001), A > D (p < 0.001), A > E (p = 0.040), F > B (p = 0.005), F > C (p = 0.007), F > D (p = 0.015). No significant differences were observed between Groups A and F, and Groups E and F (Fig. 1).

### Effects on fasting blood sugar (FBS)

There was also a significant change in FBS from the baseline when we compared the pre-medication (mean baseline FBS) and post-medication mean weight difference for each group with other groups (F = 2.701 p = 0.032) (Table 3). Post-hoc analysis revealed a significant difference between groups A and D (p = 0.011). When the mean baseline FBS (pre-medication) of each group was compared with the post-medication FBS of each group, the mean FBS changed significantly only in Group D (p < 0.001).

**Table 3:** Group comparisons in FBS change from baseline

Group	N	Mean	Std. Error	Minimum	Maximum	
A	9	4.00	3.782	− 11	26	
B	10	− 5.70	3.916	− 28	15	F = 2.701 p = **0.032**
C	9	− 6.89	4.344	− 21	13	
D	10	− 14.20	2.210	− 27	− 2	
E	8	− 2.87	4.462	− 20	15	
F	7	− 5.57	3.046	− 19	4	

Group A: Haloperidol 5 mg/kg i.p., Group B: Haloperidol 5 mg/kg i.p. before Cannabidiol 5 mg/kg p.o., Group C: Cannabidiol 5 mg/kg p.o., Group D: Control (Distilled water) 2 ml p.o. Group E: Haloperidol decanoate 50 mg/kg i.m., Group F: Haloperidol decanoate 50 mg/kg i.m. before administration of cannabidiol 5 mg/kg p.o. Time 1 (pre-medications) Time 2 (post-medications). 1. Group A, 2. Group B, 3. Group C, 4. Group D, 5. Group E, 6. Group F.

**Fig. 2:**
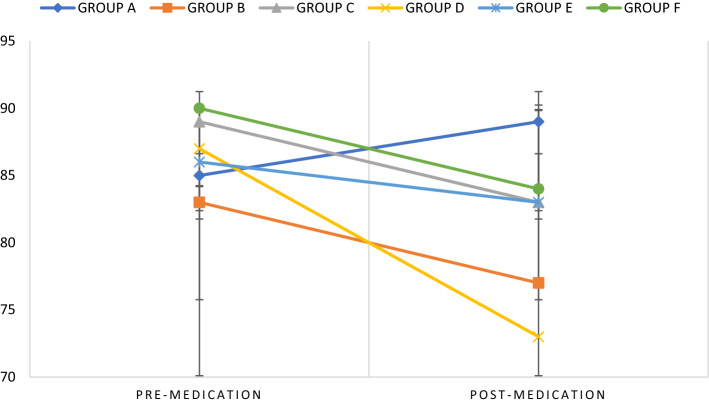
Within Subject Effects: Estimated Marginal Means of FBS over time. *Standard deviations are represented as black dashes (–) on the black vertical lines connecting the coloured lines

Pairwise comparisons of mean FBS in each group at week 0 also did not reveal any difference, but comparison of mean FBS at week 3 across groups revealed a significant difference in mean FBS between Groups A-B (p = 0.036), and Groups A-D (p < 0.001) (Fig. 2).

## Discussion

The findings in this brief report constitute one of a series of studies on the effects of CBD on subchronic and chronic haloperidol administration. We studied the effects of CBD on weight and blood glucose changes in the setting of subchronic and chronic haloperidol administration. We found significant weight gain over time in the subchronic (IP) haloperidol alone group and the chronic (IM) haloperidol before CBD group. There was also a significant within-group change in FBS after the administrations of pharmacological interventions.

The subchronic (IP) haloperidol only group (Group A) had a significantly higher weight at baseline compared to the other groups, as well as higher post-administration weight compared to other groups, with the exception of Group F, the chronic (IM) haloperidol before CBD group. This suggests that haloperidol induced weight gain in the subchronic haloperidol group only, and this may have been ameliorated by CBD in the subchronic haloperidol before CBD group (Group B). A comparison of mean weight change pre/post administration of pharmacological intervention also indicated that Group F, the chronic (IM) haloperidol before CBD group, had significant weight change compared to the rest of the groups. These observations may have been due to natural increase in weight over time, but Group E, the chronic (IM) haloperidol alone group, with the same experimental conditions as Group F (chronic (IM) haloperidol before CBD) except for the CBD administration, did not show the same pattern of weight increase as Group F. These findings suggest that CBD induced weight gain with prolonged (IM) administration of haloperidol in Group F, contrary to what was observed with subchronic haloperidol before CBD (Group B). A pairwise comparison of pre- and post- medication weight differences in each group confirmed significant increases in weight in Groups A and F. Increase in weight has been demonstrated in female rats with low dose intramuscular haloperidol administration [27]. A clinical study also observed an increase in weight in subjects on oral haloperidol 5–20 mg/day for a year [28], but studies on the effects of CBD on the observed increased in weight with prolonged administration of haloperidol are rare.

One explanation for the observations may be that persistent D_2_ receptor antagonism led to dopaminergic hypofunction in the VTA, and stimulation of hyperphagia and weight gain [3]. Elevated prolactin secretion and hyperprolactinaemia induced by prolonged dopamine receptor occupation have also been associated with increasing prevalence of obesity [3, 4, 29–31]. Dopamine agonists have also been reported to promote weight loss and may be useful in the treatment of obesity associated with hyperprolactinemia [32, 33]. D_2_ receptor antagonism may be responsible for the increase in weight we observed in subchronic (IP) haloperidol only group (Group A). CBD may have ameliorated these effects in the subchronic haloperidol group because the dopaminergic hypofunction in the VTA and hyperprolactinemia induced by dopamine receptor occupation may not have been severe enough compared to that induced by chronic and persistence receptor occupation. The endocannabinoid system (ECS) is critical in maintaining energy balance, intake, metabolism, and storage of calories; cannabinoid receptor types 1 and 2 are the two main receptors in the ECS [34–36]. CBD has been reported to reduce body weight gain in rats through its effect on CB_2_ receptors [37]. Other investigators have documented CB_2_ receptors to be important in feeding behaviour, body weight and metabolic disorders associated with obesity [38, 39]. CBD also acts as an agonist at 5HT_2A_ and as an antagonist at 5HT_3_ receptors [40] to moderate food consumption [41]. However, its agonist activity at the 5HT_1A_ receptor [42] could lead to increased food intake and weight gain [43]. This may partly explain the increase in weight observed in Group F, the chronic (IM) haloperidol before CBD group.

The serotonin system is known to inhibit dopaminergic function in the midbrain and forebrain, and serotonergic antagonists release the dopamine system from this inhibition. This said, in the presence of persistent dopaminergic blockade, the serotonergic system may not effectively inhibit the dopaminergic system. This interaction between the dopamine and serotonin systems may also partly explain why CBD did not modulate the weight gain observed in the prolonged chronic haloperidol administration group [40, 44]. However, more studies are needed to explain the interactions between CBD and weight change.

This study also found that subchronic (IP) haloperidol elevated FBS and this was ameliorated by CBD. However, CBD did not influence FBS in the chronic haloperidol groups as there was no significant difference between the chronic (IM) haloperidol alone group and chronic (IM) haloperidol group before CBD. The results also indicated that Group D (control group) showed a significant change when the individual groups’ pre- and post- medications FBS were compared, suggesting that the medications may have influenced other groups. The significant reduction in FBS from the baseline observed in the control group compared to other groups may be related to the absence of haloperidol and CBD, both of which can modify blood glucose level. It has been proposed that antipsychotics can influence metabolism in rats by increasing fasting glucose and insulin levels, probably through their effects on the pancreas, by diminishing insulin-induced insulin receptor substrate 1 (IRS-1) phosphorylation and inhibiting protein kinase 2 (Akt) activity, causing insulin resistance [4, 8, 45]. Though pancreatitis is more commonly associated with atypical antipsychotics, it is also associated with haloperidol to a lesser degree and can precede metabolic syndrome [46]. Further studies are needed to clarify the association between duration of administration and FBS changes. Haloperidol is known to induce hyperinsulinemia, which could predisposed to glucose intolerance [47, 48], and this may be responsible for the elevated FBS from baseline in the subchronic (IP) haloperidol only group, but the FBS changes from the baseline was not significant in the chronic (IM) haloperidol only group. This suggests that in addition to the duration of administration, the route of administration may have also influenced our results. The study was however not designed to explain this observation and further studies will be required to explain the role of haloperidol-induced hyperinsulinemia in FBS changes.

CBD is known to have effects on metabolism; it may alleviate hyperphagia because of its properties as a CB1 receptor antagonist/inverse agonist and 5HT_1A_ receptor agonist, and it may also promote the differentiation of adipocytes and act as an immunomodulant [9, 10, 12, 49]. CBD may also help maintain glucose homeostasis by its agonist action on G-protein-coupled receptor (GPR)55; the action may be via insulin secretion in response to glucose concentration in the serum [50]. CBD in the chronic haloperidol groups in our study may not be effective in reducing FBS, because prolonged administration of haloperidol may have more sustained adverse effects on the pancreas, leading to glucose dysregulation. An in-depth investigation of the influence of prolonged haloperidol administration on the pancreas is needed.

In summary, we demonstrated that subchronic and chronic administration of haloperidol may have effects on weight and FBS in rats, with CBD ameliorating the effects of subchronic (IP) haloperidol on FBS.

## Conclusion

Our findings suggest that if the metabolic side effects of prolonged administration of haloperidol are well established before CBD administration, CBD will have minimal effects on these side effects. Human trials are important to confirm the effectiveness of CBD on FBS and weight reduction when given early during treatment with haloperidol. Future animal and human study designs should include a D_2_ agonist and have another control group with the same treatment schedule as that of groups E and F to corroborate our findings and confirm the involvement of D_2_ receptors in weight changes observed in our study.

## Supplementary Information

Below is the link to the electronic supplementary material.

Supplementary file1 (DOCX 110 KB)

## Data Availability

The datasets generated during and/or analysed during the current study are available from the corresponding author on reasonable request.
