# Supplementary material for: Effects of cannabidiol on weight and fasting blood sugar with chronic and subchronic haloperidol administration
Source: Discov Ment Health. 2022 Jul 14;2(1):18. doi: 10.1007/s44192-022-00021-2 (PMC10501030; doi:10.1007/s44192-022-00021-2)
Supplement: Supplementary file 1 — Supplementary file1 (DOCX 110 KB) [file 44192_2022_21_MOESM1_ESM.docx]

After adjusting for the baseline weight of the animals in each group, below is the weight changes post-medication.


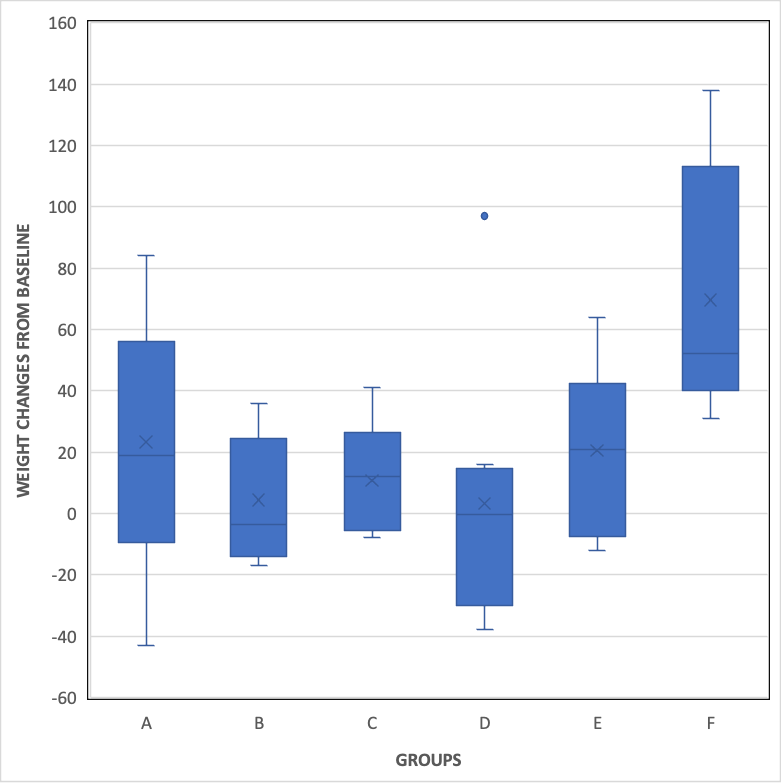


*

*

*

*Statistically significant difference between groups

After adjusting for the baseline FBS of the animals in each group, below is the FBS changes post-medication.


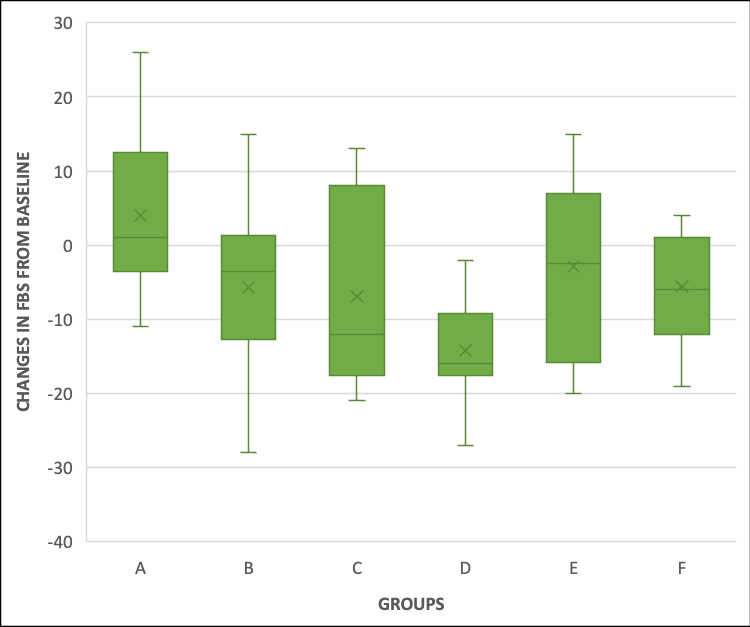


*

*Statistically significant difference between groups
